# S88. GRANDIOSE IDEAS IN SCHIZOPHRENIA: THE ROLE OF OPTIMISM BIAS AND HALLUCINATIONS

**DOI:** 10.1093/schbul/sby018.875

**Published:** 2018-04-01

**Authors:** Catherine Bortolon, Delphine Capdevielle, Hanan Yazbek, Joanna Norton, Stéphane Raffard

**Affiliations:** 1 University of Montpellier; 2 Hospital La Colombière, CHU Montpellier; 3 INSERM

## Abstract

**Background:**

Grandiose delusions (GDs) are defined as false beliefs about having an inflated worth, power, or a special identity which are firmly sustained despite undeniable evidence to the contrary. GDs have received little attention although it is the second most commonly encountered delusional beliefs (Stompe, et al., 2006). Consequently, there is no intervention program targeting GD and only few studies have explored the psychological processes underpinning GDs. Considering that delusions have an origin in autobiographical memory (Berna et al., 2017) and thus also in how individuals project themselves into the future, the aim of this present study was to explore the role of future projection, sensibility to reward and punishment, and anticipatory pleasure in GDs in schizophrenia (SZ) disorder.

**Methods:**

133 SZ patients completed measures of positive and negative symptoms, sensibility to reward and punishment, anticipatory pleasure, depression, and an experimental task in which individuals were asked to project themselves into positive, negative and neutral future situations and evaluate to what extent they believed the situation would happen in the future.

**Results:**

For the first set of analyses (Independent samples test), the sample was divided into two groups according to GD scores. Patients with higher GD scores (PANSS 5 score > 3; High GG M = 4; Low GD M = 1) obtained higher scores on sensibility to reward and self-future projection into positive situations as well as on positive symptoms. No significant differences were found regarding the other measures. Secondly, positive symptoms, sensibility to reward and positive future expectations were entered in the regression analysis. Results showed that hallucinations (B = 0.359, p = 0.0001) and positive future expectations (B = 0.216, p = 0.011) were significantly associated with GD (R2 = 0.317, p = 0.009).

**Discussion:**

This present study showed that sensibility to reward and especially higher optimism bias for the future may be important psychological processes associated with GDs in SZ patients. Optimism bias for the future may play a role in amplifying and reinforcing elated mood built upon pre-existing inflated (or accurate) perceptions of self (Freeman & Garety, 2003). Together with cognitive bias such as jumping to conclusions (Garety et al., 2012), they may provide evidence for the plausibility of GDs. Concerning the association between GDs and hallucinations, it is possible that patients experiencing hallucinations may interpret this phenomenon as a kind of special ability or power, resulting in turn in GDs maintenance.

**References:**

1. Berna, F., Evrard, R., Coutelle, R., Kobayashi, H., Laprévote, V., &Danion, J. M. (2017). Characteristics of memories of delusion-like experiences within the psychosis continuum: Pilot studies providing new insight on the relationship between self and delusions. Journal of behavior therapy and experimental psychiatry, 56, 33–41.

2. Freeman, D., & Garety, P. A. (2003). Connecting neurosis and psychosis: the direct influence of emotion on delusions and hallucinations. Behaviour research and therapy, 41(8), 923–947.

3. Garety, P. A., Gittins, M., Jolley, S., Bebbington, P., Dunn, G., Kuipers, E., ... & Freeman, D. (2012). Differences in cognitive and emotional processes between persecutory and grandiose delusions.Schizophrenia bulletin, 39(3), 629–639;

4. Stompe, T., Karakula, H., Rudalevičiene, P., Okribelashvili, N., Chaudhry, H. R., Idemudia,E. E., et al. (2006). The pathoplastic effect of culture on psychotic symptoms inschizophrenia.World Cultural Psychiatry Research Review, 157−163.

